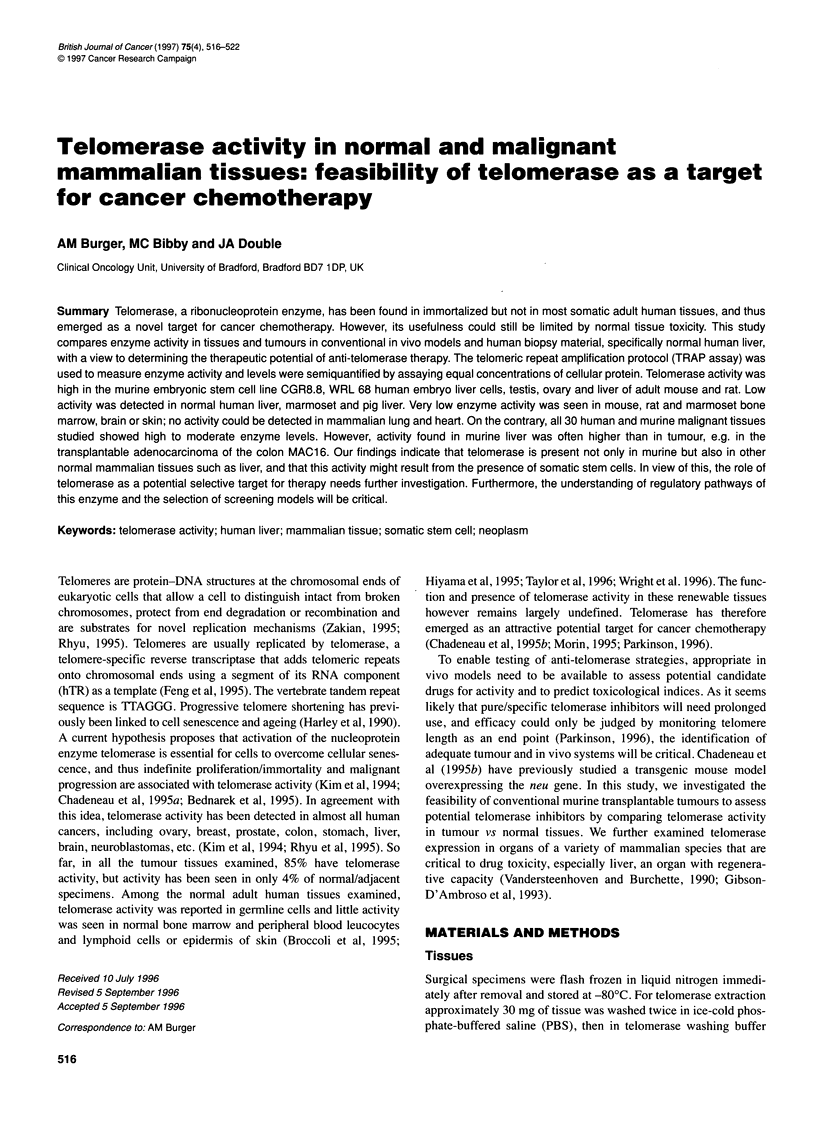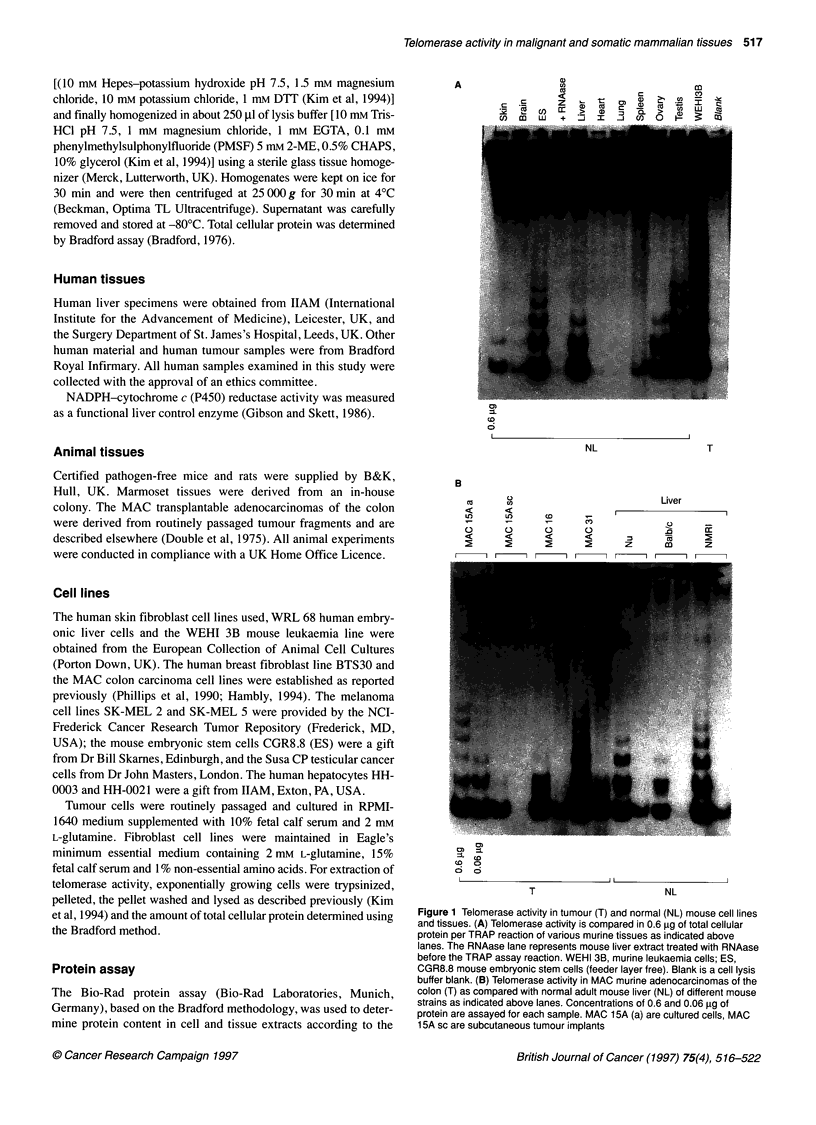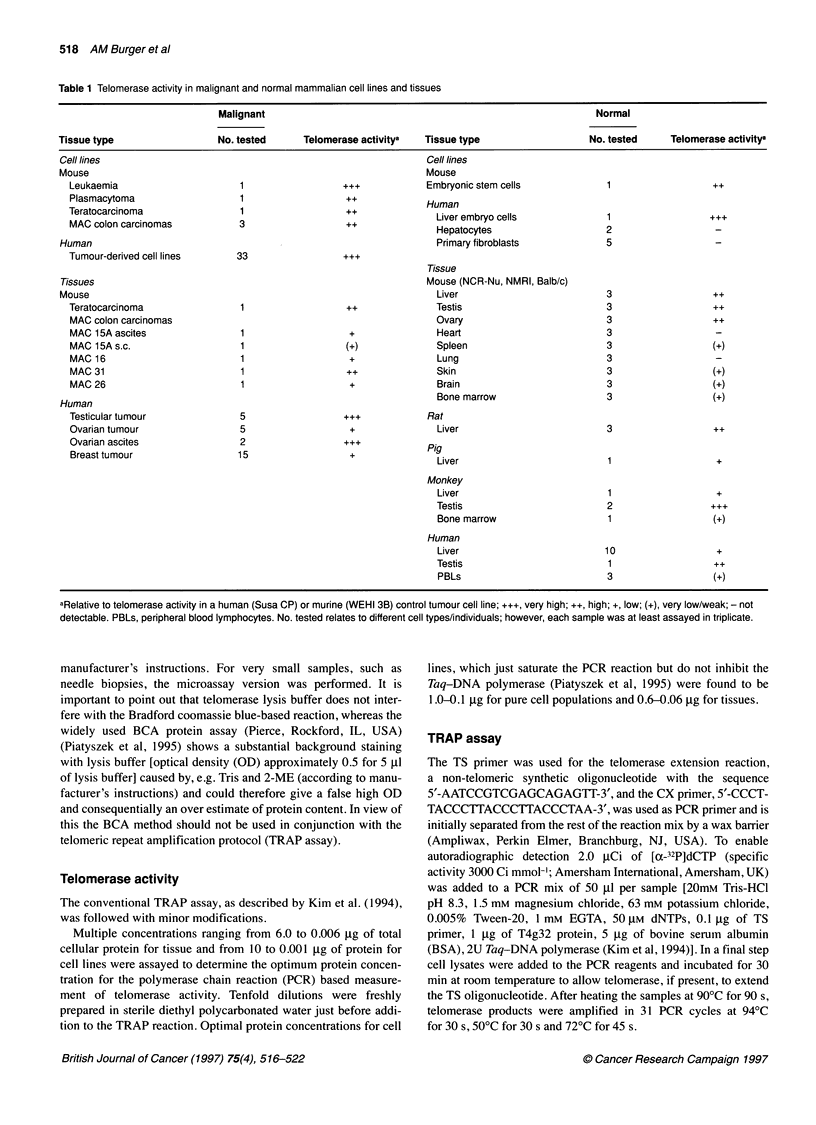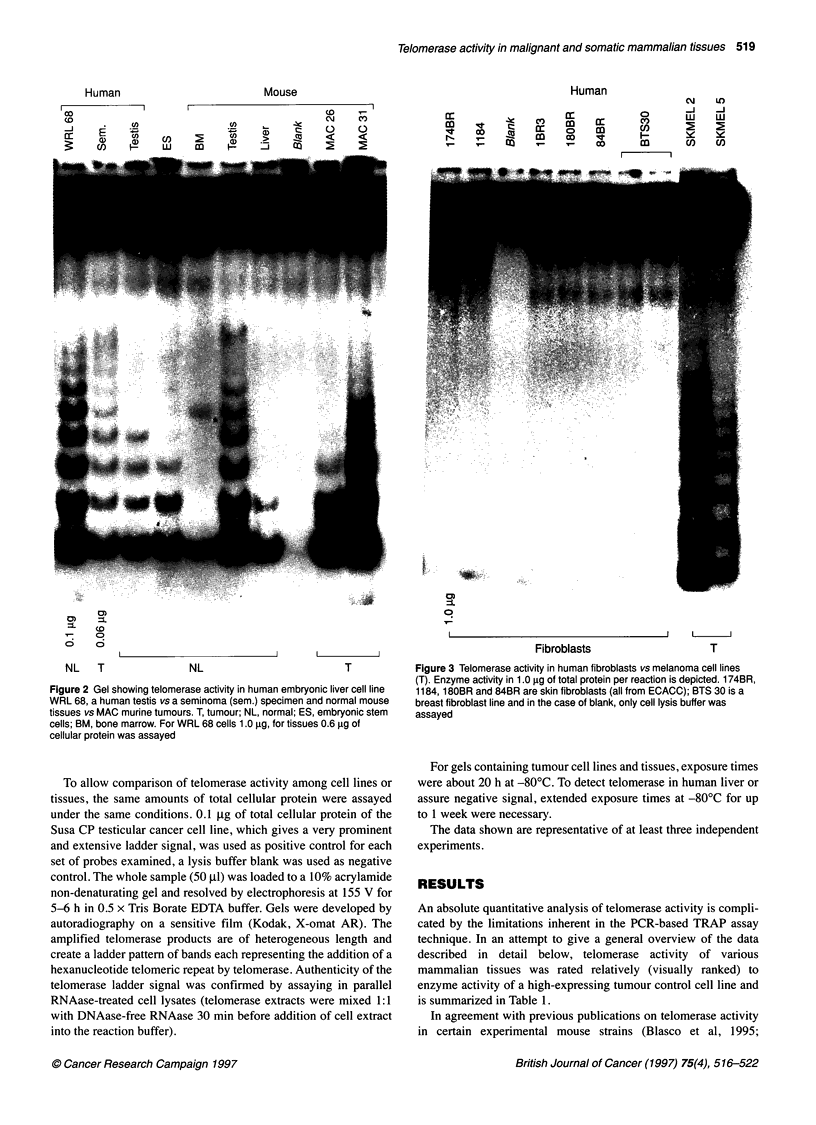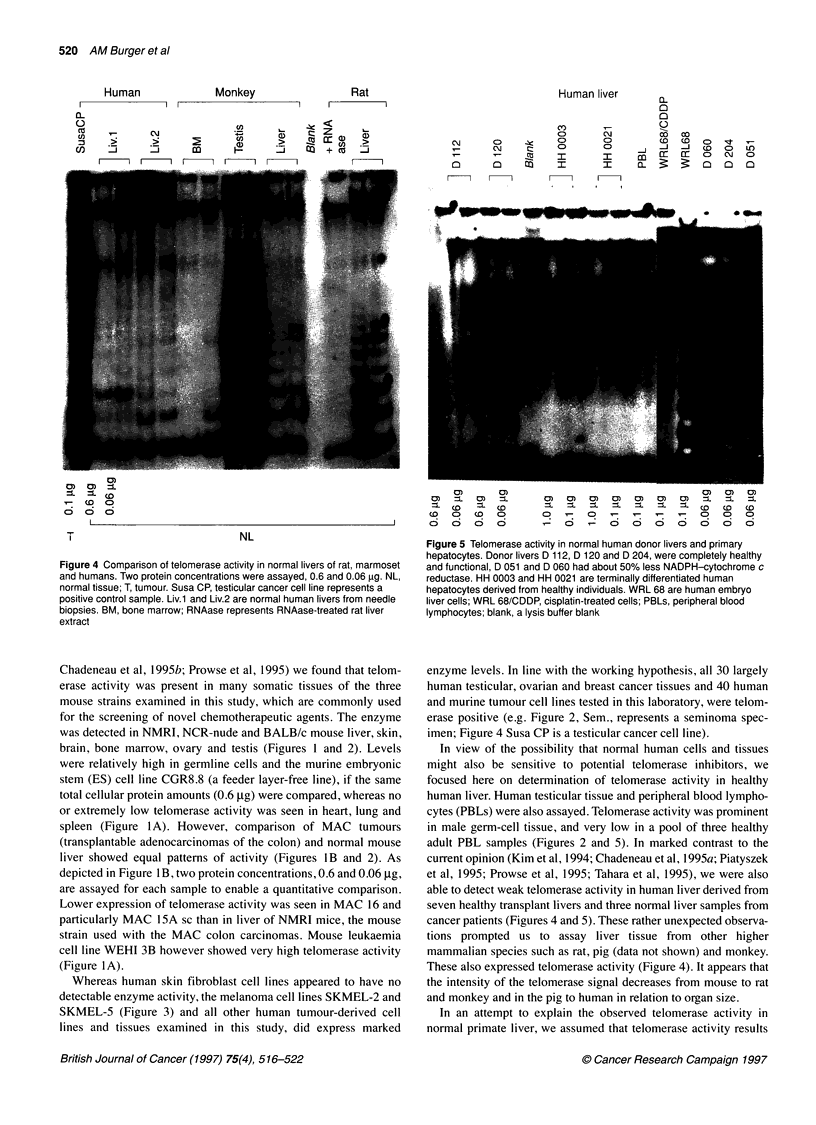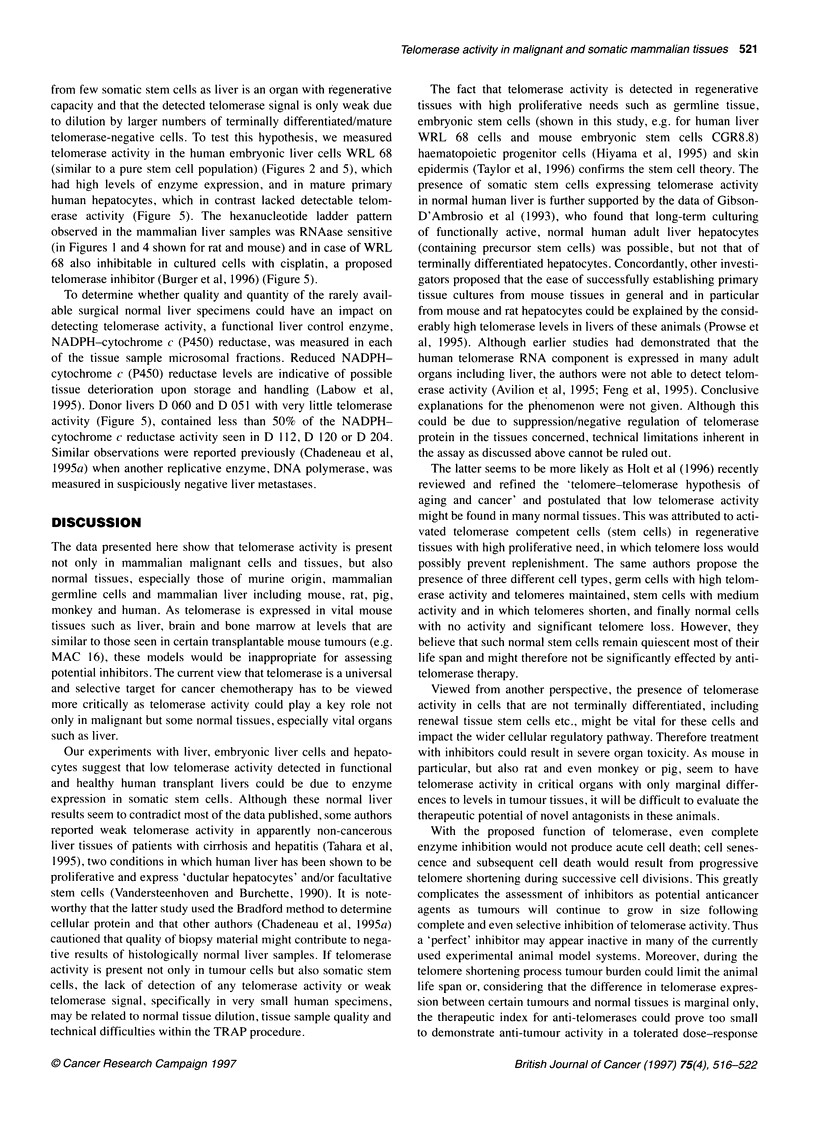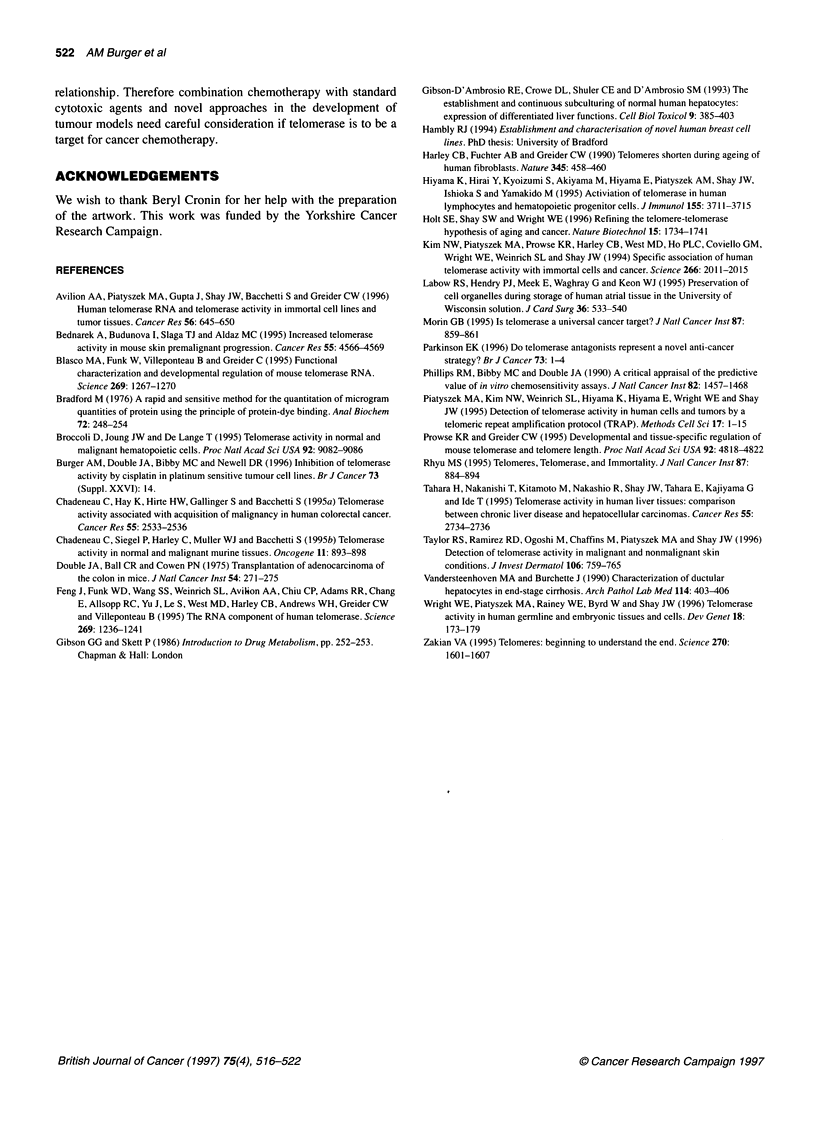# Telomerase activity in normal and malignant mammalian tissues: feasibility of telomerase as a target for cancer chemotherapy.

**DOI:** 10.1038/bjc.1997.90

**Published:** 1997

**Authors:** A. M. Burger, M. C. Bibby, J. A. Double

**Affiliations:** Clinical Oncology Unit, University of Bradford, UK.

## Abstract

**Images:**


British Journal of Cancer (1997) 75(4), 516–522
© 1997 Cancer Research Campaign

### Telomerase activity in normal and malignant mammalian tissues: feasibility of telomerase as a target for cancer chemotherapy

**AM Burger, MC Bibby and JA Double**

Clinical Oncology Unit, University of Bradford, Bradford BD7 1DP, UK

**Summary** Telomerase, a ribonucleoprotein enzyme, has been found in immortalized but not in most somatic adult human tissues, and thus emerged as a novel target for cancer chemotherapy. However, its usefulness could still be limited by normal tissue toxicity. This study compares enzyme activity in tissues and tumours in conventional in vivo models and human biopsy material, specifically normal human liver, with a view to determining the therapeutic potential of anti-telomerase therapy. The telomeric repeat amplification protocol (TRAP assay) was used to measure enzyme activity and levels were semiquantified by assaying equal concentrations of cellular protein. Telomerase activity was high in the murine embryonic stem cell line CGR8.8, WRL 68 human embryo liver cells, testis, ovary and liver of adult mouse and rat. Low activity was detected in normal human liver, marmoset and pig liver. Very low enzyme activity was seen in mouse, rat and marmoset bone marrow, brain or skin; no activity could be detected in mammalian lung and heart. On the contrary, all 30 human and murine malignant tissues studied showed high to moderate enzyme levels. However, activity found in murine liver was often higher than in tumour, e.g. in the transplantable adenocarcinoma of the colon MAC16. Our findings indicate that telomerase is present not only in murine but also in other normal mammalian tissues such as liver, and that this activity might result from the presence of somatic stem cells. In view of this, the role of telomerase as a potential selective target for therapy needs further investigation. Furthermore, the understanding of regulatory pathways of this enzyme and the selection of screening models will be critical.

**Keywords:** telomerase activity; human liver; mammalian tissue; somatic stem cell; neoplasm

Telomeres are protein–DNA structures at the chromosomal ends of eukaryotic cells that allow a cell to distinguish intact from broken chromosomes, protect from end degradation or recombination and are substrates for novel replication mechanisms (Zakian, 1995; Rhyu, 1995). Telomeres are usually replicated by telomerase, a telomere-specific reverse transcriptase that adds telomeric repeats onto chromosomal ends using a segment of its RNA component (hTR) as a template (Feng et al, 1995). The vertebrate tandem repeat sequence is TTAGGG. Progressive telomere shortening has previously been linked to cell senescence and ageing (Harley et al, 1990). A current hypothesis proposes that activation of the nucleoprotein enzyme telomerase is essential for cells to overcome cellular senescence, and thus indefinite proliferation/immortality and malignant progression are associated with telomerase activity (Kim et al, 1994; Chadeneau et al, 1995*a*; Bednarek et al, 1995). In agreement with this idea, telomerase activity has been detected in almost all human cancers, including ovary, breast, prostate, colon, stomach, liver, brain, neuroblastomas, etc. (Kim et al, 1994; Rhyu et al, 1995). So far, in all the tumour tissues examined, 85% have telomerase activity, but activity has been seen in only 4% of normal/adjacent specimens. Among the normal adult human tissues examined, telomerase activity was reported in germline cells and little activity was seen in normal bone marrow and peripheral blood leucocytes and lymphoid cells or epidermis of skin (Broccoli et al, 1995; Hiyama et al, 1995; Taylor et al, 1996; Wright et al. 1996). The function and presence of telomerase activity in these renewable tissues however remains largely undefined. Telomerase has therefore emerged as an attractive potential target for cancer chemotherapy (Chadeneau et al, 1995*b*; Morin, 1995; Parkinson, 1996).

To enable testing of anti-telomerase strategies, appropriate in vivo models need to be available to assess potential candidate drugs for activity and to predict toxicological indices. As it seems likely that pure/specific telomerase inhibitors will need prolonged use, and efficacy could only be judged by monitoring telomere length as an end point (Parkinson, 1996), the identification of adequate tumour and in vivo systems will be critical. Chadeneau et al (1995*b*) have previously studied a transgenic mouse model overexpressing the *neu* gene. In this study, we investigated the feasibility of conventional murine transplantable tumours to assess potential telomerase inhibitors by comparing telomerase activity in tumour *vs* normal tissues. We further examined telomerase expression in organs of a variety of mammalian species that are critical to drug toxicity, especially liver, an organ with regenerative capacity (Vandersteenhoven and Burchette, 1990; Gibson-D'Ambroso et al, 1993).

### MATERIALS AND METHODS

### Tissues

Surgical specimens were flash frozen in liquid nitrogen immediately after removal and stored at –80°C. For telomerase extraction approximately 30 mg of tissue was washed twice in ice-cold phosphate-buffered saline (PBS), then in telomerase washing buffer

*Received 10 July 1996*
*Revised 5 September 1996*
*Accepted 5 September 1996*

*Correspondence to:* AM Burger

[(10 mM Hepes–potassium hydroxide pH 7.5, 1.5 mM magnesium chloride, 10 mM potassium chloride, 1 mM DTT (Kim et al, 1994)] and finally homogenized in about 250 µl of lysis buffer [10 mM Tris-HCl pH 7.5, 1 mM magnesium chloride, 1 mM EGTA, 0.1 mM phenylmethylsulphonylfluoride (PMSF) 5 mM 2-ME, 0.5% CHAPS, 10% glycerol (Kim et al, 1994)] using a sterile glass tissue homogenizer (Merck, Lutterworth, UK). Homogenates were kept on ice for 30 min and were then centrifuged at 25 000 ***g*** for 30 min at 4°C (Beckman, Optima TL Ultracentrifuge). Supernatant was carefully removed and stored at –80°C. Total cellular protein was determined by Bradford assay (Bradford, 1976).

### Human tissues

Human liver specimens were obtained from IIAM (International Institute for the Advancement of Medicine), Leicester, UK, and the Surgery Department of St. James's Hospital, Leeds, UK. Other human material and human tumour samples were from Bradford Royal Infirmary. All human samples examined in this study were collected with the approval of an ethics committee.

NADPH–cytochrome *c* (P450) reductase activity was measured as a functional liver control enzyme (Gibson and Skett, 1986).

### Animal tissues

Certified pathogen-free mice and rats were supplied by B&K, Hull, UK. Marmoset tissues were derived from an in-house colony. The MAC transplantable adenocarcinomas of the colon were derived from routinely passaged tumour fragments and are described elsewhere (Double et al, 1975). All animal experiments were conducted in compliance with a UK Home Office Licence.

### Cell lines

The human skin fibroblast cell lines used, WRL 68 human embryonic liver cells and the WEHI 3B mouse leukaemia line were obtained from the European Collection of Animal Cell Cultures (Porton Down, UK). The human breast fibroblast line BTS30 and the MAC colon carcinoma cell lines were established as reported previously (Phillips et al, 1990; Hambly, 1994). The melanoma cell lines SK-MEL 2 and SK-MEL 5 were provided by the NCI-Frederick Cancer Research Tumor Repository (Frederick, MD, USA); the mouse embryonic stem cells CGR8.8 (ES) were a gift from Dr Bill Skarnes, Edinburgh, and the Susa CP testicular cancer cells from Dr John Masters, London. The human hepatocytes HH-0003 and HH-0021 were a gift from IIAM, Exton, PA, USA.

Tumour cells were routinely passaged and cultured in RPMI-1640 medium supplemented with 10% fetal calf serum and 2 mM L-glutamine. Fibroblast cell lines were maintained in Eagle's minimum essential medium containing 2 mM L-glutamine, 15% fetal calf serum and 1% non-essential amino acids. For extraction of telomerase activity, exponentially growing cells were trypsinized, pelleted, the pellet washed and lysed as described previously (Kim et al, 1994) and the amount of total cellular protein determined using the Bradford method.

### Protein assay

The Bio-Rad protein assay (Bio-Rad Laboratories, Munich, Germany), based on the Bradford methodology, was used to determine protein content in cell and tissue extracts according to the

**Figure 1** Telomerase activity in tumour (T) and normal (NL) mouse cell lines and tissues. (**A**) Telomerase activity is compared in 0.6 µg of total cellular protein per TRAP reaction of various murine tissues as indicated above lanes. The RNAase lane represents mouse liver extract treated with RNAase before the TRAP assay reaction. WEHI 3B, murine leukaemia cells; ES, CGR8.8 mouse embryonic stem cells (feeder layer free). Blank is a cell lysis buffer blank. (**B**) Telomerase activity in MAC murine adenocarcinomas of the colon (T) as compared with normal adult mouse liver (NL) of different mouse strains as indicated above lanes. Concentrations of 0.6 and 0.06 µg of protein are assayed for each sample. MAC 15A (a) are cultured cells, MAC 15A sc are subcutaneous tumour implants

*© Cancer Research Campaign 1997*

**Table 1** Telomerase activity in malignant and normal mammalian cell lines and tissues

| Malignant | | | Normal | | |
|---|---|---|---|---|---|
| Tissue type | No. tested | Telomerase activity[a] | Tissue type | No. tested | Telomerase activity[a] |
| *Cell lines* | | | *Cell lines* | | |
| Mouse | | | Mouse | | |
| Leukaemia | 1 | +++ | Embryonic stem cells | 1 | ++ |
| Plasmacytoma | 1 | ++ | *Human* | | |
| Teratocarcinoma | 1 | ++ | Liver embryo cells | 1 | +++ |
| MAC colon carcinomas | 3 | ++ | Hepatocytes | 2 | – |
| *Human* | | | Primary fibroblasts | 5 | – |
| Tumour-derived cell lines | 33 | +++ | | | |
| | | | *Tissue* | | |
| *Tissues* | | | Mouse (NCR-Nu, NMRI, Balb/c) | | |
| Mouse | | | Liver | 3 | ++ |
| Teratocarcinoma | 1 | ++ | Testis | 3 | ++ |
| MAC colon carcinomas | | | Ovary | 3 | ++ |
| MAC 15A ascites | 1 | + | Heart | 3 | – |
| MAC 15A s.c. | 1 | (+) | Spleen | 3 | (+) |
| MAC 16 | 1 | + | Lung | 3 | – |
| MAC 31 | 1 | ++ | Skin | 3 | (+) |
| MAC 26 | 1 | + | Brain | 3 | (+) |
| *Human* | | | Bone marrow | 3 | (+) |
| Testicular tumour | 5 | +++ | *Rat* | | |
| Ovarian tumour | 5 | + | Liver | 3 | ++ |
| Ovarian ascites | 2 | +++ | *Pig* | | |
| Breast tumour | 15 | + | Liver | 1 | + |
| | | | *Monkey* | | |
| | | | Liver | 1 | + |
| | | | Testis | 2 | +++ |
| | | | Bone marrow | 1 | (+) |
| | | | *Human* | | |
| | | | Liver | 10 | + |
| | | | Testis | 1 | ++ |
| | | | PBLs | 3 | (+) |

[a]Relative to telomerase activity in a human (Susa CP) or murine (WEHI 3B) control tumour cell line; +++, very high; ++, high; +, low; (+), very low/weak; – not detectable. PBLs, peripheral blood lymphocytes. No. tested relates to different cell types/individuals; however, each sample was at least assayed in triplicate.

manufacturer's instructions. For very small samples, such as needle biopsies, the microassay version was performed. It is important to point out that telomerase lysis buffer does not interfere with the Bradford coomassie blue-based reaction, whereas the widely used BCA protein assay (Pierce, Rockford, IL, USA) (Piatyszek et al, 1995) shows a substantial background staining with lysis buffer [optical density (OD) approximately 0.5 for 5 μl of lysis buffer] caused by, e.g. Tris and 2-ME (according to manufacturer's instructions) and could therefore give a false high OD and consequentially an over estimate of protein content. In view of this the BCA method should not be used in conjunction with the telomeric repeat amplification protocol (TRAP assay).

### Telomerase activity

The conventional TRAP assay, as described by Kim et al. (1994), was followed with minor modifications.

Multiple concentrations ranging from 6.0 to 0.006 μg of total cellular protein for tissue and from 10 to 0.001 μg of protein for cell lines were assayed to determine the optimum protein concentration for the polymerase chain reaction (PCR) based measurement of telomerase activity. Tenfold dilutions were freshly prepared in sterile diethyl polycarbonated water just before addition to the TRAP reaction. Optimal protein concentrations for cell lines, which just saturate the PCR reaction but do not inhibit the *Taq*–DNA polymerase (Piatyszek et al, 1995) were found to be 1.0–0.1 μg for pure cell populations and 0.6–0.06 μg for tissues.

### TRAP assay

The TS primer was used for the telomerase extension reaction, a non-telomeric synthetic oligonucleotide with the sequence 5′-AATCCGTCGAGCAGAGTT-3′, and the CX primer, 5′-CCCT-TACCCTTACCCTTACCCTAA-3′, was used as PCR primer and is initially separated from the rest of the reaction mix by a wax barrier (Ampliwax, Perkin Elmer, Branchburg, NJ, USA). To enable autoradiographic detection 2.0 μCi of [α-$^{32}$P]dCTP (specific activity 3000 Ci $mmol^{-1}$; Amersham International, Amersham, UK) was added to a PCR mix of 50 μl per sample [20mM Tris-HCl pH 8.3, 1.5 mM magnesium chloride, 63 mM potassium chloride, 0.005% Tween-20, 1 mM EGTA, 50 μM dNTPs, 0.1 μg of TS primer, 1 μg of T4g32 protein, 5 μg of bovine serum albumin (BSA), 2U *Taq*–DNA polymerase (Kim et al, 1994)]. In a final step cell lysates were added to the PCR reagents and incubated for 30 min at room temperature to allow telomerase, if present, to extend the TS oligonucleotide. After heating the samples at 90°C for 90 s, telomerase products were amplified in 31 PCR cycles at 94°C for 30 s, 50°C for 30 s and 72°C for 45 s.

© Cancer Research Campaign 1997

**Figure 2** Gel showing telomerase activity in human embryonic liver cell line WRL 68, a human testis *vs* a seminoma (sem.) specimen and normal mouse tissues *vs* MAC murine tumours. T, tumour; NL, normal; ES, embryonic stem cells; BM, bone marrow. For WRL 68 cells 1.0 µg, for tissues 0.6 µg of cellular protein was assayed

**Figure 3** Telomerase activity in human fibroblasts *vs* melanoma cell lines (T). Enzyme activity in 1.0 µg of total protein per reaction is depicted. 174BR, 1184, 180BR and 84BR are skin fibroblasts (all from ECACC); BTS 30 is a breast fibroblast line and in the case of blank, only cell lysis buffer was assayed

To allow comparison of telomerase activity among cell lines or tissues, the same amounts of total cellular protein were assayed under the same conditions. 0.1 µg of total cellular protein of the Susa CP testicular cancer cell line, which gives a very prominent and extensive ladder signal, was used as positive control for each set of probes examined, a lysis buffer blank was used as negative control. The whole sample (50 µl) was loaded to a 10% acrylamide non-denaturating gel and resolved by electrophoresis at 155 V for 5–6 h in 0.5 × Tris Borate EDTA buffer. Gels were developed by autoradiography on a sensitive film (Kodak, X-omat AR). The amplified telomerase products are of heterogeneous length and create a ladder pattern of bands each representing the addition of a hexanucleotide telomeric repeat by telomerase. Authenticity of the telomerase ladder signal was confirmed by assaying in parallel RNAase-treated cell lysates (telomerase extracts were mixed 1:1 with DNAase-free RNAase 30 min before addition of cell extract into the reaction buffer).

For gels containing tumour cell lines and tissues, exposure times were about 20 h at –80°C. To detect telomerase in human liver or assure negative signal, extended exposure times at –80°C for up to 1 week were necessary.

The data shown are representative of at least three independent experiments.

### RESULTS

An absolute quantitative analysis of telomerase activity is complicated by the limitations inherent in the PCR-based TRAP assay technique. In an attempt to give a general overview of the data described in detail below, telomerase activity of various mammalian tissues was rated relatively (visually ranked) to enzyme activity of a high-expressing tumour control cell line and is summarized in Table 1.

In agreement with previous publications on telomerase activity in certain experimental mouse strains (Blasco et al, 1995;

© Cancer Research Campaign 1997

**Figure 4** Comparison of telomerase activity in normal livers of rat, marmoset and humans. Two protein concentrations were assayed, 0.6 and 0.06 µg. NL, normal tissue; T, tumour. Susa CP, testicular cancer cell line represents a positive control sample. Liv.1 and Liv.2 are normal human livers from needle biopsies. BM, bone marrow; RNAase represents RNAase-treated rat liver extract

**Figure 5** Telomerase activity in normal human donor livers and primary hepatocytes. Donor livers D 112, D 120 and D 204, were completely healthy and functional, D 051 and D 060 had about 50% less NADPH–cytochrome *c* reductase. HH 0003 and HH 0021 are terminally differentiated human hepatocytes derived from healthy individuals. WRL 68 are human embryo liver cells; WRL 68/CDDP, cisplatin-treated cells; PBLs, peripheral blood lymphocytes; blank, a lysis buffer blank

Chadeneau et al, 1995*b*; Prowse et al, 1995) we found that telomerase activity was present in many somatic tissues of the three mouse strains examined in this study, which are commonly used for the screening of novel chemotherapeutic agents. The enzyme was detected in NMRI, NCR-nude and BALB/c mouse liver, skin, brain, bone marrow, ovary and testis (Figures 1 and 2). Levels were relatively high in germline cells and the murine embryonic stem (ES) cell line CGR8.8 (a feeder layer-free line), if the same total cellular protein amounts (0.6 µg) were compared, whereas no or extremely low telomerase activity was seen in heart, lung and spleen (Figure 1A). However, comparison of MAC tumours (transplantable adenocarcinomas of the colon) and normal mouse liver showed equal patterns of activity (Figures 1B and 2). As depicted in Figure 1B, two protein concentrations, 0.6 and 0.06 µg, are assayed for each sample to enable a quantitative comparison. Lower expression of telomerase activity was seen in MAC 16 and particularly MAC 15A sc than in liver of NMRI mice, the mouse strain used with the MAC colon carcinomas. Mouse leukaemia cell line WEHI 3B however showed very high telomerase activity (Figure 1A).

Whereas human skin fibroblast cell lines appeared to have no detectable enzyme activity, the melanoma cell lines SKMEL-2 and SKMEL-5 (Figure 3) and all other human tumour-derived cell lines and tissues examined in this study, did express marked enzyme levels. In line with the working hypothesis, all 30 largely human testicular, ovarian and breast cancer tissues and 40 human and murine tumour cell lines tested in this laboratory, were telomerase positive (e.g. Figure 2, Sem., represents a seminoma specimen; Figure 4 Susa CP is a testicular cancer cell line).

In view of the possibility that normal human cells and tissues might also be sensitive to potential telomerase inhibitors, we focused here on determination of telomerase activity in healthy human liver. Human testicular tissue and peripheral blood lymphocytes (PBLs) were also assayed. Telomerase activity was prominent in male germ-cell tissue, and very low in a pool of three healthy adult PBL samples (Figures 2 and 5). In marked contrast to the current opinion (Kim et al, 1994; Chadeneau et al, 1995*a*; Piatyszek et al, 1995; Prowse et al, 1995; Tahara et al, 1995), we were also able to detect weak telomerase activity in human liver derived from seven healthy transplant livers and three normal liver samples from cancer patients (Figures 4 and 5). These rather unexpected observations prompted us to assay liver tissue from other higher mammalian species such as rat, pig (data not shown) and monkey. These also expressed telomerase activity (Figure 4). It appears that the intensity of the telomerase signal decreases from mouse to rat and monkey and in the pig to human in relation to organ size.

In an attempt to explain the observed telomerase activity in normal primate liver, we assumed that telomerase activity results

© Cancer Research Campaign 1997

from few somatic stem cells as liver is an organ with regenerative capacity and that the detected telomerase signal is only weak due to dilution by larger numbers of terminally differentiated/mature telomerase-negative cells. To test this hypothesis, we measured telomerase activity in the human embryonic liver cells WRL 68 (similar to a pure stem cell population) (Figures 2 and 5), which had high levels of enzyme expression, and in mature primary human hepatocytes, which in contrast lacked detectable telomerase activity (Figure 5). The hexanucleotide ladder pattern observed in the mammalian liver samples was RNAase sensitive (in Figures 1 and 4 shown for rat and mouse) and in case of WRL 68 also inhibitable in cultured cells with cisplatin, a proposed telomerase inhibitor (Burger et al, 1996) (Figure 5).

To determine whether quality and quantity of the rarely available surgical normal liver specimens could have an impact on detecting telomerase activity, a functional liver control enzyme, NADPH–cytochrome *c* (P450) reductase, was measured in each of the tissue sample microsomal fractions. Reduced NADPH–cytochrome *c* (P450) reductase levels are indicative of possible tissue deterioration upon storage and handling (Labow et al, 1995). Donor livers D 060 and D 051 with very little telomerase activity (Figure 5), contained less than 50% of the NADPH–cytochrome *c* reductase activity seen in D 112, D 120 or D 204. Similar observations were reported previously (Chadeneau et al, 1995*a*) when another replicative enzyme, DNA polymerase, was measured in suspiciously negative liver metastases.

### DISCUSSION

The data presented here show that telomerase activity is present not only in mammalian malignant cells and tissues, but also normal tissues, especially those of murine origin, mammalian germline cells and mammalian liver including mouse, rat, pig, monkey and human. As telomerase is expressed in vital mouse tissues such as liver, brain and bone marrow at levels that are similar to those seen in certain transplantable mouse tumours (e.g. MAC 16), these models would be inappropriate for assessing potential inhibitors. The current view that telomerase is a universal and selective target for cancer chemotherapy has to be viewed more critically as telomerase activity could play a key role not only in malignant but some normal tissues, especially vital organs such as liver.

Our experiments with liver, embryonic liver cells and hepatocytes suggest that low telomerase activity detected in functional and healthy human transplant livers could be due to enzyme expression in somatic stem cells. Although these normal liver results seem to contradict most of the data published, some authors reported weak telomerase activity in apparently non-cancerous liver tissues of patients with cirrhosis and hepatitis (Tahara et al, 1995), two conditions in which human liver has been shown to be proliferative and express 'ductular hepatocytes' and/or facultative stem cells (Vandersteenhoven and Burchette, 1990). It is noteworthy that the latter study used the Bradford method to determine cellular protein and that other authors (Chadeneau et al, 1995*a*) cautioned that quality of biopsy material might contribute to negative results of histologically normal liver samples. If telomerase activity is present not only in tumour cells but also somatic stem cells, the lack of detection of any telomerase activity or weak telomerase signal, specifically in very small human specimens, may be related to normal tissue dilution, tissue sample quality and technical difficulties within the TRAP procedure.

The fact that telomerase activity is detected in regenerative tissues with high proliferative needs such as germline tissue, embryonic stem cells (shown in this study, e.g. for human liver WRL 68 cells and mouse embryonic stem cells CGR8.8) haematopoietic progenitor cells (Hiyama et al, 1995) and skin epidermis (Taylor et al, 1996) confirms the stem cell theory. The presence of somatic stem cells expressing telomerase activity in normal human liver is further supported by the data of Gibson-D'Ambrosio et al (1993), who found that long-term culturing of functionally active, normal human adult liver hepatocytes (containing precursor stem cells) was possible, but not that of terminally differentiated hepatocytes. Concordantly, other investigators proposed that the ease of successfully establishing primary tissue cultures from mouse tissues in general and in particular from mouse and rat hepatocytes could be explained by the considerably high telomerase levels in livers of these animals (Prowse et al, 1995). Although earlier studies had demonstrated that the human telomerase RNA component is expressed in many adult organs including liver, the authors were not able to detect telomerase activity (Avilion et al, 1995; Feng et al, 1995). Conclusive explanations for the phenomenon were not given. Although this could be due to suppression/negative regulation of telomerase protein in the tissues concerned, technical limitations inherent in the assay as discussed above cannot be ruled out.

The latter seems to be more likely as Holt et al (1996) recently reviewed and refined the 'telomere–telomerase hypothesis of aging and cancer' and postulated that low telomerase activity might be found in many normal tissues. This was attributed to activated telomerase competent cells (stem cells) in regenerative tissues with high proliferative need, in which telomere loss would possibly prevent replenishment. The same authors propose the presence of three different cell types, germ cells with high telomerase activity and telomeres maintained, stem cells with medium activity and in which telomeres shorten, and finally normal cells with no activity and significant telomere loss. However, they believe that such normal stem cells remain quiescent most of their life span and might therefore not be significantly effected by anti-telomerase therapy.

Viewed from another perspective, the presence of telomerase activity in cells that are not terminally differentiated, including renewal tissue stem cells etc., might be vital for these cells and impact the wider cellular regulatory pathway. Therefore treatment with inhibitors could result in severe organ toxicity. As mouse in particular, but also rat and even monkey or pig, seem to have telomerase activity in critical organs with only marginal differences to levels in tumour tissues, it will be difficult to evaluate the therapeutic potential of novel antagonists in these animals.

With the proposed function of telomerase, even complete enzyme inhibition would not produce acute cell death; cell senescence and subsequent cell death would result from progressive telomere shortening during successive cell divisions. This greatly complicates the assessment of inhibitors as potential anticancer agents as tumours will continue to grow in size following complete and even selective inhibition of telomerase activity. Thus a 'perfect' inhibitor may appear inactive in many of the currently used experimental animal model systems. Moreover, during the telomere shortening process tumour burden could limit the animal life span or, considering that the difference in telomerase expression between certain tumours and normal tissues is marginal only, the therapeutic index for anti-telomerases could prove too small to demonstrate anti-tumour activity in a tolerated dose–response

© Cancer Research Campaign 1997

relationship. Therefore combination chemotherapy with standard cytotoxic agents and novel approaches in the development of tumour models need careful consideration if telomerase is to be a target for cancer chemotherapy.

### ACKNOWLEDGEMENTS

We wish to thank Beryl Cronin for her help with the preparation of the artwork. This work was funded by the Yorkshire Cancer Research Campaign.

### REFERENCES

Avilion AA, Piatyszek MA, Gupta J, Shay JW, Bacchetti S and Greider CW (1996) Human telomerase RNA and telomerase activity in immortal cell lines and tumor tissues. *Cancer Res* **56**: 645–650

Bednarek A, Budunova I, Slaga TJ and Aldaz MC (1995) Increased telomerase activity in mouse skin premalignant progression. *Cancer Res* **55**: 4566–4569

Blasco MA, Funk W, Villeponteau B and Greider C (1995) Functional characterization and developmental regulation of mouse telomerase RNA. *Science* **269**: 1267–1270

Bradford M (1976) A rapid and sensitive method for the quantitation of microgram quantities of protein using the principle of protein-dye binding. *Anal Biochem* **72**: 248–254

Broccoli D, Joung JW and De Lange T (1995) Telomerase activity in normal and malignant hematopoietic cells. *Proc Natl Acad Sci USA* **92**: 9082–9086

Burger AM, Double JA, Bibby MC and Newell DR (1996) Inhibition of telomerase activity by cisplatin in platinum sensitive tumour cell lines. *Br J Cancer* **73** (Suppl. XXVI): 14.

Chadeneau C, Hay K, Hirte HW, Gallinger S and Bacchetti S (1995*a*) Telomerase activity associated with acquisition of malignancy in human colorectal cancer. *Cancer Res* **55**: 2533–2536

Chadeneau C, Siegel P, Harley C, Muller WJ and Bacchetti S (1995*b*) Telomerase activity in normal and malignant murine tissues. *Oncogene* **11**: 893–898

Double JA, Ball CR and Cowen PN (1975) Transplantation of adenocarcinoma of the colon in mice. *J Natl Cancer Inst* **54**: 271–275

Feng J, Funk WD, Wang SS, Weinrich SL, Avilion AA, Chiu CP, Adams RR, Chang E, Allsopp RC, Yu J, Le S, West MD, Harley CB, Andrews WH, Greider CW and Villeponteau B (1995) The RNA component of human telomerase. *Science* **269**: 1236–1241

Gibson GG and Skett P (1986) *Introduction to Drug Metabolism*, pp. 252–253. Chapman & Hall: London

Gibson-D'Ambrosio RE, Crowe DL, Shuler CE and D'Ambrosio SM (1993) The establishment and continuous subculturing of normal human hepatocytes: expression of differentiated liver functions. *Cell Biol Toxicol* **9**: 385–403

Hambly RJ (1994) *Establishment and characterisation of novel human breast cell lines*. PhD thesis: University of Bradford

Harley CB, Fuchter AB and Greider CW (1990) Telomeres shorten during ageing of human fibroblasts. *Nature* **345**: 458–460

Hiyama K, Hirai Y, Kyoizumi S, Akiyama M, Hiyama E, Piatyszek AM, Shay JW, Ishioka S and Yamakido M (1995) Activiation of telomerase in human lymphocytes and hematopoietic progenitor cells. *J Immunol* **155**: 3711–3715

Holt SE, Shay SW and Wright WE (1996) Refining the telomere-telomerase hypothesis of aging and cancer. *Nature Biotechnol* **15**: 1734–1741

Kim NW, Piatyszek MA, Prowse KR, Harley CB, West MD, Ho PLC, Coviello GM, Wright WE, Weinrich SL and Shay JW (1994) Specific association of human telomerase activity with immortal cells and cancer. *Science* **266**: 2011–2015

Labow RS, Hendry PJ, Meek E, Waghray G and Keon WJ (1995) Preservation of cell organelles during storage of human atrial tissue in the University of Wisconsin solution. *J Card Surg* **36**: 533–540

Morin GB (1995) Is telomerase a universal cancer target? *J Natl Cancer Inst* **87**: 859–861

Parkinson EK (1996) Do telomerase antagonists represent a novel anti-cancer strategy? *Br J Cancer* **73**: 1–4

Phillips RM, Bibby MC and Double JA (1990) A critical appraisal of the predictive value of *in vitro* chemosensitivity assays. *J Natl Cancer Inst* **82**: 1457–1468

Piatyszek MA, Kim NW, Weinrich SL, Hiyama K, Hiyama E, Wright WE and Shay JW (1995) Detection of telomerase activity in human cells and tumors by a telomeric repeat amplification protocol (TRAP). *Methods Cell Sci* **17**: 1–15

Prowse KR and Greider CW (1995) Developmental and tissue-specific regulation of mouse telomerase and telomere length. *Proc Natl Acad Sci USA* **92**: 4818–4822

Rhyu MS (1995) Telomeres, Telomerase, and Immortality. *J Natl Cancer Inst* **87**: 884–894

Tahara H, Nakanishi T, Kitamoto M, Nakashio R, Shay JW, Tahara E, Kajiyama G and Ide T (1995) Telomerase activity in human liver tissues: comparison between chronic liver disease and hepatocellular carcinomas. *Cancer Res* **55**: 2734–2736

Taylor RS, Ramirez RD, Ogoshi M, Chaffins M, Piatyszek MA and Shay JW (1996) Detection of telomerase activity in malignant and nonmalignant skin conditions. *J Invest Dermatol* **106**: 759–765

Vandersteenhoven MA and Burchette J (1990) Characterization of ductular hepatocytes in end-stage cirrhosis. *Arch Pathol Lab Med* **114**: 403–406

Wright WE, Piatyszek MA, Rainey WE, Byrd W and Shay JW (1996) Telomerase activity in human germline and embryonic tissues and cells. *Dev Genet* **18**: 173–179

Zakian VA (1995) Telomeres: beginning to understand the end. *Science* **270**: 1601–1607

© Cancer Research Campaign 1997